# Expression and Localization of Ferritin-Heavy Chain Predicts Recurrence for Breast Cancer Patients with a *BRCA1/2* Mutation

**DOI:** 10.3390/cancers16010028

**Published:** 2023-12-20

**Authors:** Shuoying Qu, A. Mieke Timmermans, Bernadette A. M. Heemskerk-Gerritsen, Anita M. A. C. Trapman-Jansen, Renée Broeren-Foekens, Wendy J. C. Prager-van der Smissen, Hoesna El Hassnaoui, Tim van Tienhoven, Claudia K. Bes-Stobbe, Pieter J. Westenend, Carolien H. M. van Deurzen, John W. M. Martens, Maartje J. Hooning, Antoinette Hollestelle

**Affiliations:** 1 Department of Medical Oncology, Erasmus MC Cancer Institute, Erasmus University Medical Center, 3015 GD Rotterdam, The Netherlands; 2 Pathan BV, Kleiweg 500, 3045 PM Rotterdam, The Netherlands; 3 Laboratory of Pathology, 3318 AL Dordrecht, The Netherlands; 4 Department of Pathology, Erasmus University Medical Center, 3000 CA Rotterdam, The Netherlands

**Keywords:** *BRCA1*, *BRCA2*, germline mutation, breast cancer, survival, FTH1, T-cell response

## Abstract

**Simple Summary:**

Ferritin is a ferroxidase, which protects cellular components from the potentially toxic effects of free iron. The expression and localization of ferritin-heavy chain (FTH1), the catalytic subunit of ferritin, was shown to predict survival for triple-negative breast cancer (BC) patients and be related to T-cell response. Here, we studied the association between FTH1 and time to survival in primary BCs from 222 *BRCA1/2* mutation carriers. We found that nuclear, but not cytoplasmic, localization of FTH1 expression was associated with a shorter time to recurrence. In a subset of 51 *BRCA1/2* mutation carriers, we evaluated the relation between localization and expression of FTH1 and T-cell response. However, we did not detect any association between FTH1 and the amount or composition of CD8+ cytotoxic, CD4+ helper, or FOXP3+ regulatory T cells. Further research is necessary to unravel the mechanism by which nuclear FTH1 influences the clinical outcome of *BRCA1/2*-associated BC patients.

**Abstract:**

The ferritin-heavy chain (FTH1) is the catalytic subunit of the ferroxidase ferritin, which prevents oxidative DNA damage via intracellular iron storage. FTH1 was shown to be a prognostic marker for triple-negative breast cancer (BC) patients and associated with an enrichment of CD8+ effector T cells. However, whether the expression and localization of FTH1 are also associated with clinical outcome in other BC subtypes is unknown. Here, we investigated the association of FTH1 with time to survival in BCs from 222 *BRCA1/2* mutation carriers by immunohistochemistry on tissue microarrays. In addition, for 51 of these patients, the association between FTH1 and specific subsets of T cells was evaluated on whole slides using automatic scoring algorithms. We revealed that nuclear FTH1 (nFTH1) expression, in multivariable analyses, was associated with a shorter disease-free (HR = 2.71, 95% CI = 1.49–4.92, *p* = 0.001) and metastasis-free survival (HR = 3.54, 95% CI = 1.45–8.66, *p* = 0.006) in patients carrying a *BRCA1/2* mutation. However, we found no relation between cytoplasmic FTH1 expression and survival of *BRCA1/2* mutation carriers. Moreover, we did not detect an association between FTH1 expression and the amount of CD45+ (*p* = 0.13), CD8+ (*p* = 0.18), CD4+ (*p* = 0.20) or FOXP3+ cells (*p* = 0.17). Consequently, the mechanism underlying the worse recurrence-free survival of nFTH1 expression in *BRCA1/2* mutation carriers needs further investigation.

## 1. Introduction

Ferritin is an iron-binding protein that is present in both the intra- and extracellular compartments. It mainly functions as a ferroxidase in iron sequestration, capturing and converting ferrous iron (Fe^2+^) into ferric iron (Fe^3+^). Ferritin is essential for iron homeostasis by making iron available for critical cellular processes, such as oxygen transport or conversion of oxygen into usable cellular energy. On the other hand, ferritin protects cellular components such as DNA and proteins from the potentially toxic effects of free iron, which can directly generate reactive oxygen species (ROS) via the Fenton reaction. The latter induces ferroptosis, a lipid peroxidation-driven and iron-dependent form of regulated cell death. Non-canonical induction of ferroptosis may occur through decreased ferritin expression, for example, through NCOA4-mediated ferritin autophagy. In addition, an excess of iron has also been linked to an altered distribution of T-cell subsets by increasing and decreasing the number and activity of CD8+ cytotoxic T cells and CD4+ helper T cells, respectively. Moreover, excess iron also alters the anti-tumor action of monocytes and macrophages [1,2,3,4].

The ferritin protein is made up of 24 subunits of two different subtypes: ferritin-heavy chains (FTH1; 21 kD) and ferritin-light chains (FTL; 18.5 kD) [5]. The ratio of FTH1, which possesses the ferroxidase catalytic activity, to FTL in the ferritin protein varies widely among tissue types and can be modulated under inflammatory or infectious circumstances [1]. Interestingly, tissue ferritin levels are significantly increased in breast cancer (BC) compared to normal or benign breast tumor tissue [6,7]. Moreover, both FTH1 and FTL expression have been associated with clinical outcomes of BC patients. FTL was shown to be associated with shorter metastasis-free survival (MFS) in node-negative BC patients [8]. However, in a study by Liu et al. FTL was not associated with MFS, while FTH1 was associated with longer MFS in triple-negative BC (TNBC) patients [9].

Previous research has also indicated that the subcellular localization of ferritin is relevant. Particularly in aggressive spindle-like TNBC cell lines, which displayed elevated levels of FTH1 and FTL compared with epithelial BC cell lines, increased ferritin was localized in the nucleus [10]. In line with this, Liu et al. showed that TNBC patients with high (>1%) nuclear FTH1 (nFTH1) expression had a higher risk of metastasis, while high (>75%) cytoplasmic FTH1 (cFTH1) was associated with lower risk of metastasis in these patients [11]. Intriguingly, high expression of cFTH1 was linked to an increase in CD8+ effector T cells but not CD4+ helper T cells. Finally, activation of the CXCL12-CXCR4 signaling pathway was shown to induce time-dependent cFTH1 to nFTH1 switching [12]. Concordantly, CXCL12-CXCR4 signaling promotes migration, invasion, and metastasis of malignant BC cells [13,14].

Around 10% of BC patients have a family history of the disease. In this respect, *BRCA1* and *BRCA2* are the two most prevalently mutated and penetrant BC genes. In a cancer genetic clinic-based setting, *BRCA1* and *BRCA2* mutation carriers have a cumulative BC risk of 71% and 64% at 70 years old, while this is 65% and 45% at 70 years in a cohort unselected for family history [15,16]. Importantly, patients with a *BRCA1* mutation mostly develop TNBC, while BCs of *BRCA2* mutation carriers are generally ER+ [17]. Moreover, BRCA1 and BRCA2-deficient BCs are unable to adequately repair DNA double-strand breaks through the homologous recombination pathway, rendering these tumors highly sensitive to DNA-damaging agents, such as interstrand crosslinking agents, topo-isomerase II inhibitors or PARP inhibitors [18,19,20]. So far, a few studies have indicated either a direct or indirect link between *BRCA1*, PARP inhibition, and ferroptosis [21,22,23].

By using immunohistochemistry on tissue microarrays, we evaluated whether subcellular localization and expression of FTH1 in 222 BCs of *BRCA1/2* mutation carriers were associated with clinical outcome after BC. In addition, we also analyzed whether cFTH1 and nFTH1 expression were correlated with different subsets of T cells in these BCs using an automated scoring algorithm.

## 2. Materials and Methods

### 2.1. Study Population

We retrieved primary formalin-fixed paraffin-embedded (FFPE) breast tumor blocks from 241 *BRCA1/2* mutation carriers who were counseled at our Clinical Genetic Center and from whom we had previously collected clinical data for survival analyses, as well as treatment data and clinicopathological variables. Tumor blocks were retrieved from the pathology lab at the Erasmus University Medical Center as well as pathology labs from 12 other Dutch hospitals. Patients were diagnosed with BC between 1982 and 2008 and received genetic testing for *BRCA1/2* genes between 1996 and 2008. In 191 patients (79.3%), a pathogenic *BRCA1* germline mutation was detected, whereas 50 (20.7%) patients carried a pathogenic *BRCA2* germline mutation. This study was approved by the Medical Ethics Committee of the Erasmus University Medical Center (MEC 02-953).

### 2.2. Tissue Microarray Generation

Primary FFPE tumor blocks were sectioned, and H&E stains were prepared. The invasive tumor area was marked on these H&E sections, and tumor grade and histology were reevaluated by a breast pathologist (CvD). Tissue microarray blocks were constructed by punching three 0.6 mm cores from the invasive tumor area from each patient’s primary tumor block and placing these in three different donor blocks using a TMA Grand Master (3DHisTech, Budapest, Hungary). This way, three donor TMA blocks were generated, all including one core from each patient, but organized in a different order.

### 2.3. Immunohistochemical Staining

Tissue microarray blocks were sectioned and placed on SuperFrost Plus slides (Fisher Scientific, Waltham, MA, USA). After deparaffination and dehydration of sections, antigen retrieval was performed by boiling sections for 40 min in DAKO Target Retrieval Solution (TRS) pH = 6 using a water bath (Glostrup, Denmark). Next, sections were blocked for endogenous peroxidase with 0.3% H_2_O_2_ in PBS and additionally blocked using 5% BSA in PBS. The primary antibody against FTH1 (rabbit anti-human FTH1, clone EPR3005Y; Genetex, Irvine, CA, USA) was diluted 1:100 in DAKO Antibody Diluent and incubated for 1 h at room temperature. FTH1 expression was visualized using the rabbit Envision+ system (DAKO), and nuclei were counterstained with hematoxylin.

FFPE blocks from 51 of the 241 *BRCA1/2* (42 *BRCA1* and 9 *BRCA2*) mutation carriers that were retrieved from the Erasmus University Medical Center were additionally stained for leukocyte marker CD45, cytotoxic T-cell marker CD8, helper T-cell marker CD4, and regulatory T-cell marker FOXP3 on whole sections, as described above. Antibodies were mouse anti-human CD45 clone 2B11 and PD7/26 (Cell Marque (Rocklin, CA, USA); TRS pH = 8, ready-to-use), mouse anti-human CD8 clone C8/144B (DAKO; TRS pH = 9, 1:100), mouse anti-human CD4 clone 4B12 (DAKO; TRS pH = 9, 1:80), and mouse anti-human FOXP3 clone 236A/E7 (Abcam (Cambridge, UK); TRS pH = 6, 1:50).

### 2.4. Manual and Automated Scoring

**Figure 1:**
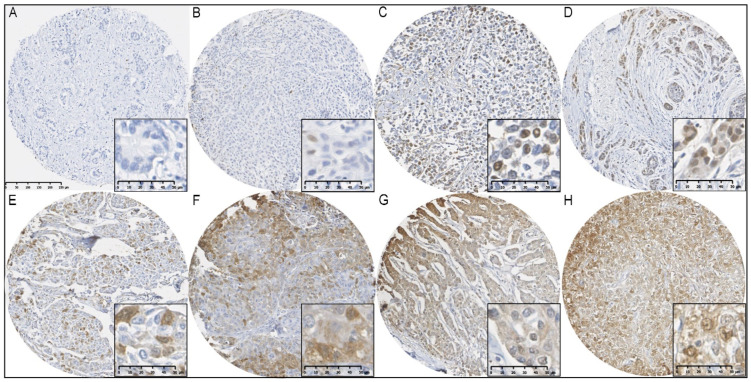
Immunohistochemical staining of FTH1 in BCs from *BRCA1/2* mutation carriers. (**A**) FTH1 negative; (**B**) ≤1% nuclear FTH1 expression; (**C**,**D**) >1% nuclear FTH1 expression; (**E**) ≤75% cytoplasmic FTH1 expression; (**F**,**G**) >75% cytoplasmic FTH1 expression; (**H**) nuclear and cytoplasmic FTH1 expression.

Tissue microarray and whole slides were digitized using the Nanozoomer 2 digital slide scanner (Hamamatsu, Bridgewater, NJ, USA). Tissue microarray scans were uploaded to the Distiller TMA software v2.2 (Leica Microsystems, Wetzlar, Germany) and manually scored for cytoplasmic as well as nuclear FTH1 staining by quantifying the percentage of positively stained invasive tumor cells (Figure 1). Cytoplasmic FTH1 scores were dichotomized into low (≤75%) and high (>75%), whereas nuclear FTH1 scores were dichotomized into negative (≤1%) and positive (>1%), according to Liu et al. [11]. For 19 out of the 241 *BRCA1/2* mutation carriers, we were unable to quantify the nFTH1 and cFTH1 scores from the breast tumor tissues due to lost or folded cores or cores not containing invasive tumor cells. Therefore, all analyses were performed with a maximum total of 222 *BRCA1/2* mutation carriers, of whom 178 were *BRCA1* and 44 were *BRCA2* mutation carriers. The patient characteristics of these 222 *BRCA1/2* mutation carriers can be found in Table 1.

Whole slide scans were uploaded to QuPath v0.2.0, and the invasive tumor area was annotated. The percentages of CD45+, CD8+, CD4+, and FOXP3+ cells from the total number of cells in the annotated region were quantified using the positive cell detection algorithm. For all parameters except background radius (6 µm), sigma (1.65 µm), and cell expansion (4 µm), we used standard settings for this algorithm. In addition, we quantified the composition of the three different types of T cells by using the following ratios: CD8+/CD45+; CD4+/CD45+; CD4+/CD8+; and FOXP3+/CD4+.

### 2.5. Statistical Analysis

To evaluate the association between dichotomized FTH1 expression and clinicopathological factors, we performed a χ^2^ test or a Fisher’s Exact test when expected values in one of the groups were smaller than five. Because the majority of patients (87.1%) had their genetic DNA test after BC diagnosis, we accounted for potential survival bias by performing left-truncated survival analysis for disease-free, metastasis-free, overall, and BC-specific survival [24]. Consequently, time at risk started at the date of BC diagnosis or DNA test result, whichever came last, and ended either at the date of an event or censoring. This did not change the moment the event occurred but modified the time at risk until the event occurred. Events for DFS included contralateral BCs, local recurrences, lymph node metastases, or distant metastases, while for MFS, only distant metastases were considered. For OS, we considered death from all causes, whereas only death from BC was considered an event for BCSS. Censoring events were a secondary non-BC malignancy or end of follow-up time. Survival probabilities were plotted using the Kaplan–Meier method, and differences between survival curves were evaluated using the logrank test. Cox proportional hazard models were used to calculate hazard ratios (HRs) and 95% confidence intervals (CIs), and *p*-values were from the Wald test. Evaluation of proportional hazard assumptions was performed via plotting the Schoenfeld residuals. Multivariable Cox regression models included all clinicopathological values that were associated with survival time in the univariable model. Associations between dichotomized FTH1 expression and the amount and composition of T cells were evaluated using the Wilcoxon rank sum test with continuity correction. All reported *p*-values are two-sided.

## 3. Results

### 3.1. Clinicopathological Variables

The patient cohort consisted of 222 BC patients, of whom 178 were *BRCA1* and 44 were *BRCA2* mutation carriers. As expected, BCs from *BRCA1* mutation carriers were more frequently of medullary histology and ER and PR negative. Moreover, BCs from these patients more often had a poor differentiation grade compared with *BRCA2* mutation carriers (Table 1). For these 222 BC patients with a germline *BRCA1* or *BRCA2* mutation, we analyzed the association of cFTH1 (Table 2) and nFTH1 (Table 3) expression with the relevant clinicopathological variables. The data showed that cFTH1 expression levels were lower in tumors from patients diagnosed after 2000 in all mutation carriers (*p* = 0.033) and *BRCA1* mutation carriers (*p* = 0.028) but not *BRCA2* mutation carriers. In addition, *BRCA2* carriers with ER- BC had a higher cFTH1 (*p* < 0.001; Table 2).

*BRCA1/2* mutation carriers whose tumors displayed expression of nFTH1 more frequently had a favorable tumor grade (*p* = 0.015) as well as positive ER (*p* = 0.002) and PR status (*p* = 0.029). Moreover, for *BRCA1* mutation carriers, nFTH1 expression in the tumor was associated with smaller tumor size (*p* = 0.045) and positive HER2 status (*p* = 0.049), while in *BRCA2* mutation carriers, nFTH1 expression was associated with positive ER status (*p* = 0.004; Table 3).

### 3.2. Survival Analysis

**Figure 2:**
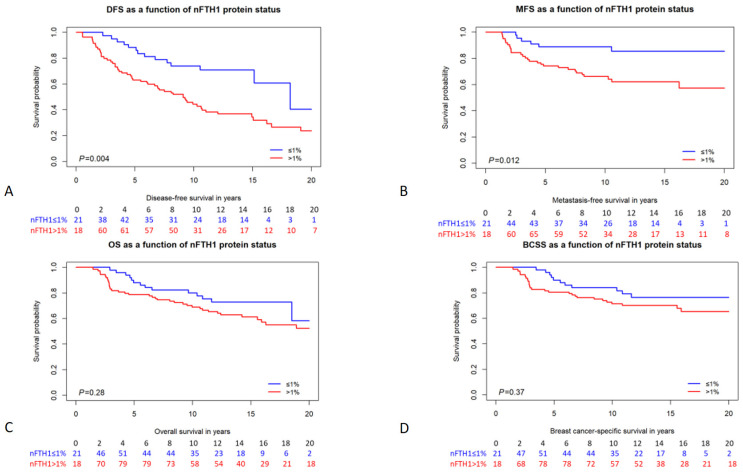
Kaplan–Meier survival curves of *BRCA1/2* mutation carriers stratified by nFTH1 expression. (**A**) disease-free survival; (**B**) metastasis-free survival; (**C**) overall survival; (**D**) breast cancer-specific survival. The number of persons at risk per time point is indicated below each graph. *p*-values are from the logrank test.

We analyzed the association of FTH1 localization and expression with different survival parameters, including disease-free survival (DFS), MFS, overall survival (OS), and BC-specific survival (BCSS). In patients carrying a germline *BRCA1/2* mutation, cFTH1 expression was not associated with any of the survival parameters in Cox proportional hazards analysis (DFS HR = 1.17, 95% CI = 0.72–1.90, *p* = 0.53; MFS HR = 1.10, 95% CI = 0.55–2.21, *p* = 0.79; OS HR = 1.09, 95% CI = 0.62–1.91, *p* = 0.78; BCSS HR = 1.17, 95% CI = 0.61–2.25, *p* = 0.64; Table 4). Also, when analyzing *BRCA1* and *BRCA2* mutation carriers separately, cFTH1 expression was not associated with time to survival in either of the two subgroups (Table 4). We also analyzed the relationship between the expression of nFTH1 and the time to survival in patients carrying *BRCA1/2* mutations. Interestingly, we found nFTH1 expression to be associated with a shorter DFS and MFS in both univariable (DFS HR = 2.32, 95% CI = 1.29–4.20, *p* = 0.005; MFS HR = 2.94, 95% CI = 1.21–7.12, *p* = 0.017; Figure 2 and Table 4) and multivariable analyses (DFS HR = 2.71, 95% CI = 1.49–4.92, *p* = 0.001; MFS HR = 3.54, 95% CI = 1.45–8.66, *p* = 0.006; Table 4). However, OS (HR = 1.40, 95% CI = 0.75–2.61, *p* = 0.28) and BCSS (HR = 1.38, 95% CI = 0.68–2.80, *p* = 0.37) were not associated with nFTH1 expression in *BRCA1/2* mutation carriers (Figure 2 and Table 4). Because their clinicopathological characteristics differ considerably, we also performed subanalyses in *BRCA1* and *BRCA2* mutation carriers separately. Consistent with the results in all mutation carriers, nFTH1 expression was associated with a shorter DFS and MFS in both univariable (DFS HR = 2.29, 95% CI = 1.20–4.39, *p* = 0.012; MFS HR = 3.14, 95% CI = 1.18–8.39, *p* = 0.022) and multivariable (DFS HR = 3.02, 95% CI = 1.54–5.91, *p* = 0.001; MFS HR = 4.47, 95% CI = 1.62–12.3, *p* = 0.004) analyses in *BRCA1* mutation carriers, but not with differences in OS and BCSS (Supplementary Figure S1 and Table 4). However, we found no association between nFTH1 and survival in *BRCA2* mutation carriers (Supplementary Figure S1 and Table 4).

### 3.3. Immune Cells

In 2014, Liu et al. showed that high cFTH1 expression was associated with a favorable prognosis and a clearly decreased ratio between CD4+ and CD8+ T cells in TNBC. In addition, they hypothesized that the decreased CD4+ T-cell density in cFTH1 high TNBC tumors could be related to a lower density of FOXP3+ regulatory T cells [11]. Although we only found an association between nFTH1, not cFTH1, expression, and clinical outcome in *BRCA1/2* mutation carriers, we still aimed to verify whether T-cell response played a role in this. Therefore, we determined the percentages of CD45+, CD8+, CD4+, and FOXP3+ cells in whole slides of BCs from 51 *BRCA1/2* mutation carriers using an automated scoring algorithm. In this cohort, we did not find any association between cFTH1 expression and the amount (CD45+ *p* = 0.32, CD8+ *p* = 0.34, CD4+ *p* = 0.93, FOXP3+ *p* = 0.48) or composition (CD8+/CD45+ *p* = 0.33, CD4+/CD45+ *p* = 0.80, CD4+/CD8+ *p* = 0.41, FOXP3+/CD4+ *p* = 0.63) of the various immune cells. Similarly, we did not find any relation between the amount (CD45+ *p* = 0.13, CD8+ *p* = 0.18, CD4+ *p* = 0.20, FOXP3+ *p* = 0.17) or composition (CD8+/CD45+ *p* = 0.91, CD4+/CD45+ *p* = 0.98, CD4+/CD8+ *p* = 0.84, FOXP3+/CD4+ *p* = 1) of the various immune cells and nFTH1 expression.

## 4. Discussion

Here, we studied the expression and subcellular localization of FTH1 in BCs from 222 *BRCA1/2* mutation carriers and investigated the association with the patients’ clinicopathological variables, survival, as well as their T-cell response in a subset of 51 *BRCA1/2* mutation carriers.

We noticed that the expression of nFTH1 was associated with more favorable clinicopathological parameters in our study, including a smaller tumor size and a lower tumor grade. In addition, nFTH1-positive BCs were also more frequently ER- and PR-positive. This seems counterintuitive since we also established that nFTH1 is an independent predictor of shorter DFS and MFS. Positive associations between cFTH1 as well as nFTH1 and the clinicopathological variables, however, could have been false positive associations as a consequence of the multiple statistical tests we performed. Indeed, after conservative Bonferroni adjustment for multiple tests, only the association between high cFTH1 expression and negative ER status in *BRCA2* mutation carriers remained significant.

Based on our results in *BRCA1/2* mutation carriers, nuclear expression of FTH1 was only associated with patients’ DFS and MFS but not with OS or BCSS. It is, however, implausible that nFTH1 is associated with the recurrence of the disease but not death. Therefore, we considered that the number of events is likely higher for DFS and MFS compared with OS and BCSS, and the absence of an association with OS and BCSS might be a consequence of statistical power. In line with this, 39.0% of *BRCA1/2* mutation carriers had a DFS event, while 23.5% and 17.9% of carriers had an OS or BCSS event. However, the rate of MFS events in these patients was similar to the rate of OS and BCSS events (i.e., 18.8%), suggesting that power was likely not an issue in our analyses unless the effect size for OS and BCSS was smaller. Considering the survival probability curves of the two nFTH1 groups were closer in Figure 2 for BCSS and OS than for DFS and MFS, this might be a plausible explanation. Since we had a relatively long median follow-up time of 111 months for our cohort, it is likely that conclusive evidence requires an expansion of the number of patients rather than the follow-up time. We do have to point out, though, that numbers at risk become very low after 15 years of follow-up time.

The expression of nFTH1 was significantly associated with shorter DFS and MFS in *BRCA1/2* and *BRCA1* mutation carriers but not in *BRCA2* mutation carriers alone. This may not be entirely unexpected since nFTH1 expression was previously found to be related to worse prognosis in TNBC patients [11], and *BRCA1* mutation is generally associated with TNBC [17]. Thus, the function of FTH1 may be associated with hormone receptor status. Alternatively, the loss of genome stability in both TNBC and *BRCA1/2* mutation carriers, in combination with the deregulation of iron homeostasis, could also be the underlying mechanism. Since we were only able to include 42 *BRCA2* mutation carriers in this study, we may not have had enough power to detect an association in this group. Therefore, conclusive evidence regarding the role of nFTH1 expression in the survival of *BRCA2* mutation carriers requires a larger study population.

We did not detect any associations between cFTH1 expression and survival in *BRCA1* mutation carriers. These results conflict with the study of Liu et al., in which they showed that high expression of cFTH1 was related to a favorable prognosis in TNBC [11]. In concordance with our results, though, Liu et al. showed that nFTH1 expression was associated with an adverse prognosis. Further research has to determine whether this discrepancy is a consequence of biological differences between TNBC and BCs of *BRCA1/2* mutation carriers.

Liu et al. also suggested that high cFTH1 expression indicated a favorable prognosis via enrichment of CD8+ T cells in TNBCs, while CD4+ T cells were diminished [11]. They hypothesized that this lower density of CD4+ T cells could be related to a lower density of immune suppressive regulatory T cells. Moreover, in our data, we observed a lower percentage of medullary BCs among nFTH1-positive compared with nFTH1-negative BCs (16.9% vs. 30.1%), which suggests a lower amount of tumor-infiltrating lymphocytes among nFTH1 positive BCs. However, we do not find any relation between the expression and localization of FTH1 and the density of CD8+, CD4+, or FOXP3+ T cells, as well as the ratios between CD8+/CD45+, CD4+/CD45+, CD4+/CD8+, and FOXP3+/CD4+ cells. Compared with Liu et al.’s study, we used a more quantitative method involving automated scoring of the immune cell markers using QuPath v0.2.0 while they estimated the percentage of positively stained cells. However, this disconcordance may more likely be the result of true biological differences between TNBCs and BCs from *BRCA1/2* mutation carriers. Therefore, future research avenues should focus on the biological effects of cellular FTH1 localization and its relation to *BRCA1/2* mutation status, hormone receptor status, ferroptosis, other immune cells such as monocytes and macrophages and activation of the CXCL12-CXCR4 signaling pathway [1,2,3,4,12,21,22,23].

## 5. Conclusions

Nuclear localization of FTH1 was associated with a shorter DFS and MFS but not OS and BCSS in a cohort of 222 *BRCA1/2* mutation carriers. This association was particularly pronounced in *BRCA1* mutation carriers; however, conclusive evidence regarding this association in *BRCA2* mutation carriers is pending. We found no evidence of an association between cytoplasmic localization of FTH1 and the time to survival. Moreover, we did not find a relation between the expression and localization of FTH1 and the density or composition of CD8+ cytotoxic T cells, CD4+ helper T cells, or FOXP3+ regulatory T cells in these BCs. Therefore, the mechanism by which nFTH1 influences the clinical outcome of *BRCA1/2* mutation carriers is still unclear. Further research should focus on the biological effects of cellular FTH1 localization and its relation to *BRCA1/2* mutation status, hormone receptor status, ferroptosis, and downstream immune response.

## Figures and Tables

**Table 1:** Patient characteristics for 222 *BRCA1*/*2* mutation carriers.

	All Mutation Carriers	BRCA1 Mutation Carriers	BRCA2 Mutation Carriers	*p*-Value
Total number	222	178 (80.2%)	44 (19.8%)	
Median follow-up time (range) in years	9.2 (0.1–18.1)	9.7 (0.1–18.1)	8.3 (0.1–15.2)	
Year of diagnosis				0.053
<2000	102	88 (86.3%)	14 (13.7%)	
≥2000	120	90 (75.0%)	30 (25.0%)	
Age at diagnosis (in years)				0.23
≤35	69	60 (87.0%)	9 (13.0%)	
36–50	124	96 (77.4%)	28 (22.6%)	
>50	29	22 (75.9%)	7 (24.1%)	
Menopausal status				0.12
Premenopausal	164	129 (78.7%)	35 (21.3%)	
Postmenopausal	29	27 (93.1%)	2 (6.9%)	
Tumor size				0.72
pT1	124	101 (81.5%)	23 (18.5%)	
pT2-4 + Unknown	98	77 (78.6%)	21 (21.4%)	
Nodal status				0.019
Negative	152	129 (84.9%)	23 (15.1%)	
Positive	67	47 (70.1%)	20 (29.9%)	
Tumor grade				<0.001
Good/Moderate	35	21 (60.0%)	14 (40.0%)	
Poor	157	135 (86.0%)	22 (14.0%)	
Tumor histology				<0.001
NST	141	109 (77.3%)	32 (22.7%)	
Medullary	45	43 (95.6%)	2 (4.4%)	
Other	23	13 (56.5%)	10 (43.5%)	
ER status				<0.001
Negative	159	147 (92.5%)	12 (7.5%)	
Positive	63	31 (49.2%)	32 (50.8%)	
PR status				<0.001
Negative	176	161 (91.5%)	15 (8.5%)	
Positive	46	17 (37.0%)	29 (63.0%)	
HER2 status				1
Negative	214	171 (80.0%)	43 (20.0%)	
Positive	8	7 (87.5%)	1 (12.5%)	
Surgery				1
Lumpectomy	118	95 (80.5%)	23 (19.5%)	
Mastectomy	103	82 (79.6%)	21 (20.4%)	
Adjuvant systemic therapy				0.87
No	73	58 (79.5%)	15 (20.5%)	
Yes	148	119 (80.4%)	29 (19.6%)	

Note: If the number of patients did not add up to 222, there were missing values for these variables. NST, non-specific type.

**Table 2:** Association of cytoplasmatic FTH1 expression status with clinicopathological variables in primary breast cancers from 222 *BRCA1* or *BRCA2* mutation carriers.

Variables	All Mutation Carriers		*p*-Value	*BRCA1* Mutation Carriers		*p*-Value	*BRCA2* Mutation Carriers		*p*-Value
	cFTH1 ≤ 75%	cFTH1 > 75%		cFTH1 ≤ 75%	cFTH1 > 75%		cFTH1 ≤ 75%	cFTH1 > 75%	
Total number	106 (49.3%)	109 (50.7%)		88 (50.9%)	85 (49.1%)		18 (42.9%)	24 (57.1%)	
Median follow-up time (range) in years	9.1 (0.1–17.3)	9.5 (0.4–18.1)		9.7 (0.1–17.3)	10.0 (0.4–18.1)		8.1 (0.1–12.6)	9.2 (2.5–15.2)	
Year of diagnosis			0.033			0.028			0.74
<2000	41 (41.0%)	59 (59.0%)		36 (41.9%)	50 (58.1%)		5 (35.7%)	9 (64.3%)	
≥2000	65 (56.5%)	50 (43.5%)		52 (59.8%)	35 (40.2%)		13 (46.4%)	15 (53.6%)	
Age at diagnosis (in years)			0.47			0.52			0.24
≤35	36 (54.5%)	30 (45.5%)		33 (56.9%)	25 (43.1%)		3 (37.5%)	5 (62.5%)	
36–50	58 (48.3%)	62 (51.7%)		44 (47.3%)	49 (52.7%)		14 (51.9%)	13 (48.1%)	
>50	12 (41.4%)	17 (58.6%)		11 (50.0%)	11 (50.0%)		1 (14.3%)	6 (85.7%)	
Menopausal status			1			0.96			0.50
Premenopausal	74 (46.8%)	84 (53.2%)		59 (47.2%)	66 (52.8%)		15 (45.5%)	18 (54.5%)	
Postmenopausal	13 (46.4%)	15 (53.6%)		13 (50.0%)	13 (50.0%)		0 (0%)	2 (100%)	
Tumor size			0.52			0.84			0.50
pT1	62 (51.7%)	58 (48.3%)		51 (52.0%)	47 (48.0%)		11 (50.0%)	11 (50.0%)	
pT2-4 + Unknown	44 (46.3%)	51 (53.7%)		37 (49.3%)	38 (50.7%)		7 (35.0%)	13 (65.0%)	
Nodal status			0.31			0.32			1
Negative	76 (51.7%)	71 (48.3%)		67 (53.6%)	58 (46.4%)		9 (40.9%)	13 (59.1%)	
Positive	28 (43.1%)	37 (56.9%)		20 (43.5%)	26 (56.5%)		8 (42.1%)	11 (57.9%)	
Tumor grade			0.33			0.86			0.075
Good/Moderate	20 (60.1%)	13 (39.4%)		12 (57.1%)	9 (42.9%)		8 (66.7%)	4 (33.3%)	
Poor	75 (49.3%)	77 (50.7%)		68 (52.3%)	62 (47.7%)		7 (31.8%)	15 (68.2%)	
Tumor histology			0.86			0.96			0.53
NST	65 (48.1%)	70 (51.9%)		52 (50.0%)	52 (50.0%)		13 (41.9%)	18 (58.1%)	
Medullary	22 (48.9%)	23 (51.1%)		22 (51.2%)	21 (48.8%)		0 (0%)	2 (100%)	
Other	12 (54.5%)	10 (45.5%)		7 (53.8%)	6 (46.2%)		5 (55.6%)	4 (44.4%)	
ER status			0.24			0.77			<0.001
Negative	71 (46.4%)	82 (53.6%)		71 (50.0%)	71 (50.0%)		0 (0%)	11 (100%)	
Positive	35 (56.5%)	27 (43.5%)		17 (54.8%)	14 (45.2%)		18 (58.1%)	13 (41.9%)	
PR status			0.44			0.66			0.057
Negative	81 (47.6%)	89 (52.4%)		78 (50.0%)	78 (50.0%)		3 (21.4%)	11 (78.6%)	
Positive	25 (55.6%)	20 (44.4%)		10 (58.8%)	7 (41.2%)		15 (53.6%)	13 (46.4%)	
HER2 status			0.49			1			0.43
Negative	101 (48.8%)	106 (51.2%)		84 (50.6%)	82 (49.4%)		17 (41.5%)	24 (58.5%)	
Positive	5 (62.5%)	3 (37.5%)		4 (57.1%)	3 (42.9%)		1 (100%)	0 (0%)	
Surgery			0.69			0.87			0.82
Lumpectomy	54 (47.4%)	60 (52.6%)		45 (49.5%)	46 (50.5%)		9 (39.1%)	14 (60.9%)	
Mastectomy	51 (51.0%)	49 (49.0%)		42 (51.9%)	39 (48.1%)		9(47.4%)	10 (52.6%)	
Adjuvant systemic therapy			0.21			0.12			0.96
No	31 (42.5%)	42 (57.5%)		24 (41.4%)	34 (58.6%)		7 (46.7%)	8 (53.3%)	
Yes	74 (52.5%)	67 (47.5%)		63 (55.3%)	51 (44.7%)		11 (40.7%)	16 (59.3%)	

Note: If the number of patients did not add up to 222, there were missing values for these variables. NST, non-specific type.

**Table 3:** Association of nuclear FTH1 expression status with clinicopathological variables in primary breast cancers from 222 *BRCA1* or *BRCA2* mutation carriers.

Variables	All Mutation Carriers		*p*-Value	*BRCA1* Mutation Carriers		*p*-Value	*BRCA2* Mutation Carriers		*p*-Value
	nFTH1 ≤ 1%	nFTH1 > 1%		nFTH1 ≤ 1%	nFTH1 > 1%		nFTH1 ≤ 1%	nFTH1 > 1%	
Total number	76 (34.2%)	146 (65.8%)		65 (36.5%)	113 (63.5%)		11 (25.0%)	33 (75.0%)	
Median follow-up time (range) in years	9.9 (0.1–16.7)	9.1 (0.1–18.1)		9.7 (0.1–16.7)	9.7 (0.1–18.1)		10.4 (0.1–12.8)	8.2 (0.5–15.2)	
Year of diagnosis			0.87			1			0.72
<2000	36 (35.3%)	66 (64.7%)		32 (36.4%)	56 (63.6%)		4 (28.6%)	10 (71.4%)	
≥2000	40 (33.3%)	80 (66.7%)		33 (36.7%)	57 (63.3%)		7 (23.3%)	23 (76.7%)	
Age at diagnosis (in years)			0.18			0.24			0.53
≤35	20 (29.0%)	49 (71.0%)		18 (30.0%)	42 (70.0%)		2 (22.2%)	7 (77.8%)	
36–50	42 (33.9%)	82 (66.1%)		36 (37.5%)	60 (62.5%)		6 (21.4%)	22 (78.6%)	
>50	14 (48.2%)	15 (51.7%)		11 (50.0%)	11 (50.0%)		3 (42.9%)	4 (57.1%)	
Menopausal status			0.75			1			0.39
Premenopausal	54 (32.9%)	110 (67.1%)		47 (36.4%)	82 (63.6%)		7 (20.0%)	28 (80.0%)	
Postmenopausal	11 (37.9%)	18 (62.1%)		10 (37.0%)	17 (63.0%)		1 (50.0%)	1 (50.0%)	
Tumor size			0.26			0.045			0.17
pT1	38 (30.6%)	86 (69.4%)		30 (29.7%)	71 (70.3%)		8 (34.8%)	15 (65.2%)	
pT2-4 + Unknown	38 (38.8%)	60 (61.2%)		35 (45.5%)	42 (54.5%)		3 (14.3%)	18 (85.7%)	
Nodal status			0.40			1			0.18
Negative	56 (36.8%)	96 (63.2%)		48 (37.2%)	81 (62.8%)		8 (34.8%)	15 (65.2%)	
Positive	20 (29.9%)	47 (70.1%)		17 (36.2%)	30 (63.8%)		3 (15.0%)	17 (85.0%)	
Tumor grade			0.015			0.056			0.25
Good/Moderate	6 (17.1%)	29 (82.9%)		4 (19.0%)	17 (81.0%)		2 (14.3%)	12 (85.7%)	
Poor	64 (40.8%)	93 (59.2%)		56 (41.5%)	79 (58.5%)		8 (36.4%)	14 (63.6%)	
Tumor histology			0.074			0.15			0.55
NST	45 (31.9%)	96 (68.1%)		38 (34.9%)	71 (65.1%)		7 (21.9%)	25 (78.1%)	
Medullary	22 (48.9%)	23 (51.1%)		21 (48.8%)	22 (51.2%)		1 (50.0%)	1 (50.0%)	
Other	6 (26.1%)	17 (73.9%)		3 (23.1%)	10 (76.9%)		3 (30.0%)	7 (70.0%)	
ER status			0.002			0.12			0.004
Negative	65 (40.9%)	94 (59.1%)		58 (39.5%)	89 (60.5%)		7 (58.3%)	5 (41.7%)	
Positive	11 (17.5%)	52 (82.5%)		7 (22.6%)	24 (77.4%)		4 (12.5%)	28 (87.5%)	
PR status			0.029			0.11			0.47
Negative	67 (38.1%)	109 (61.9%)		62 (38.5%)	99 (61.5%)		5 (33.3%)	10 (66.7%)	
Positive	9 (19.6%)	37 (80.4%)		3 (17.6%)	14 (82.4%)		6 (20.7%)	23 (79.3%)	
*HER2* status			0.053			0.049			1
Negative	76 (35.5%)	138 (64.5%)		65 (38.0%)	106 (62.0%)		11 (25.6%)	32 (74.4%)	
Positive	0 (0%)	8 (100%)		0 (0%)	7 (100%)		0 (0%)	1 (100%)	
Surgery			1			0.90			0.49
Lumpectomy	41 (34.7%)	77 (65.3%)		34 (35.8%)	61 (64.2%)		7 (30.4%)	16 (69.6%)	
Mastectomy	35 (34.0%)	68 (66.0%)		31 (37.8%)	51 (62.2%)		4 (19.0%)	17 (81.0%)	
Adjuvant systemic therapy			0.28			0.054			0.14
No	21 (28.8%)	52 (71.2%)		15 (25.9%)	43 (74.1%)		6 (40.0%)	9 (60.0%)	
Yes	55 (37.2%)	93 (62.8%)		50 (42.0%)	69 (58.0%)		5 (17.2%)	24 (82.8%)	

Note: If the number of patients did not add up to 222, there were missing values for these variables. NST, non-specific type.

**Table 4:** Uni- and multivariable Cox regression analysis of 222 *BRCA1* or *BRCA2* mutation carriers by FTH1 expression and localization.

FTH1 Localization	Survival Endpoint	Analysis	All Mutation Carriers	*BRCA1* Mutation Carriers	*BRCA2* Mutation Carriers
Cytoplasmic	DFS	Univariable	N = 166, E = 66, HR = 1.17, 95% CI = 0.72–1.90, *p* = 0.53	N = 131, E = 52, HR = 1.22, 95% CI = 0.70–2.11, *p* = 0.48	N = 35, E = 14, HR = 0.99, 95% CI = 0.33–2.98, *p* = 0.99
	MFS	Univariable	N = 170, E = 32, HR = 1.10, 95% CI = 0.55–2.21, *p* = 0.79	N = 134, E = 25, HR = 1.10, 95% CI = 0.50–2.41, *p* = 0.82	N = 36, E = 7, HR = 1.05, 95% CI = 0.23–4.76, *p* = 0.95
	OS	Univariable	N = 206, E = 49, HR = 1.09, 95% CI = 0.62–1.91, *p* = 0.78	N = 167, E = 40, HR = 1.03, 95% CI = 0.55–1.93, *p* = 0.93	N = 39, E = 9, HR = 1.47, 95% CI = 0.37–5.93, *p* = 0.59
	BCSS	Univariable	N = 200, E = 36, HR = 1.17, 95% CI = 0.61–2.25, *p* = 0.64	N = 162, E = 29, HR = 1.24, 95% CI = 0.60–2.57, *p* = 0.57	N = 38, E = 7, HR = 0.98, 95% CI = 0.22–4.40, *p* = 0.97
Nuclear	DFS	Univariable	N = 172, E = 67, HR = 2.32, 95% CI = 1.29–4.20, *p* = 0.005	N = 135, E = 52, HR = 2.29, 95% CI = 1.20–4.39, *p* = 0.012	N = 37, E = 15, HR = 3.59, 95% CI = 0.79–16.24, *p* = 0.097
		Multivariable ^a^	N = 172, E = 67, HR = 2.71, 95% CI = 1.49–4.92, *p* = 0.001	N = 135, E = 52, HR = 3.02, 95% CI = 1.54–5.91, *p* = 0.001	
	MFS	Univariable	N = 176, E = 33, HR = 2.94, 95% CI = 1.21–7.12, *p* = 0.017	N = 138, E = 25, HR = 3.14, 95% CI = 1.18–8.39, *p* = 0.022	N = 38, E = 8, HR = 2.91, 95% CI = 0.35–23.98, *p* = 0.32
		Multivariable ^b^	N = 174, E = 33, HR = 3.54, 95% CI = 1.45–8.66, *p* = 0.006	N = 137, E = 25, HR = 4.47, 95% CI = 1.62–12.3, *p* = 0.004	
	OS	Univariable	N = 213, E = 50, HR = 1.40, 95% CI = 0.75–2.61, *p* = 0.28	N = 172, E = 41, HR = 1.52, 95% CI = 0.77–3.00, *p* = 0.23	N = 41, E = 9, HR = 0.94, 95% CI = 0.19–4.71, *p* = 0.94
	BCSS	Univariable	N = 207, E = 37, HR = 1.38, 95% CI = 0.68–2.80, *p* = 0.37	N = 167, E = 30, HR = 1.42, 95% CI = 0.66–3.04, *p* = 0.37	N = 40, E = 7, HR = 1.53, 95% CI = 0.18–13.22, *p* = 0.70

DFS, disease-free survival; MFS, metastasis-free survival; OS, overall survival; BCSS, breast cancer-specific survival; N, number of patients in the analysis; E, number of events; HR, hazard ratio; CI, confidence interval. *p*-values are from the Wald test. ^a^ adjusted for tumor size; ^b^ adjusted for tumor size and nodal status.

## Data Availability

Tissue microarray data are available upon request from the corresponding author.

## Supplementary Materials

The following supporting information can be downloaded at https://www.mdpi.com/article/10.3390/cancers16010028/s1. Figure S1. Kaplan–Meier survival curves of *BRCA1* and *BRCA2* mutation carriers stratified by nFTH1 expression.
