# Supplementary material for: Expression and Localization of Ferritin-Heavy Chain Predicts Recurrence for Breast Cancer Patients with a BRCA1/2 Mutation
Source: Cancers (Basel). 2023 Dec 20;16(1):28. doi: 10.3390/cancers16010028 (PMC10778040; doi:10.3390/cancers16010028)
Supplement: Supplementary file 1 [file cancers-16-00028-s001.zip › cancers-2772561-supplementary.pdf]

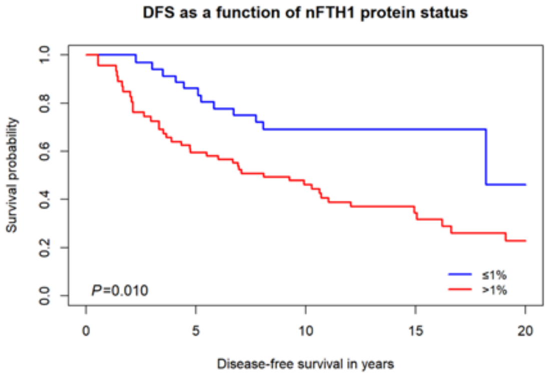

A

	0	2	4	6	8	10	12	14	16	18	20
nFTH1≤1%	30	32	35	28	25	19	14	12	4	3	1
nFTH1>1%	15	40	42	41	36	27	21	15	11	9	6

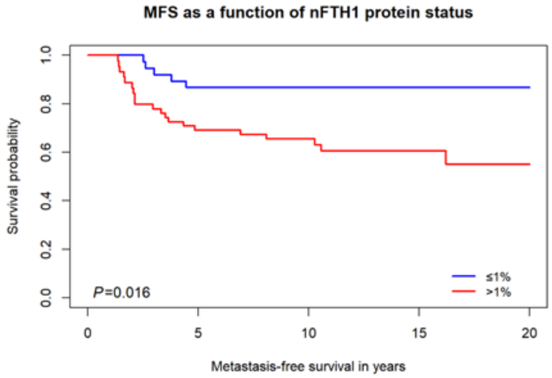

B

	0	2	4	6	8	10	12	14	16	18	20
nFTH1≤1%	30	36	36	30	28	21	14	12	4	3	1
nFTH1>1%	15	40	44	41	37	28	21	15	11	9	6

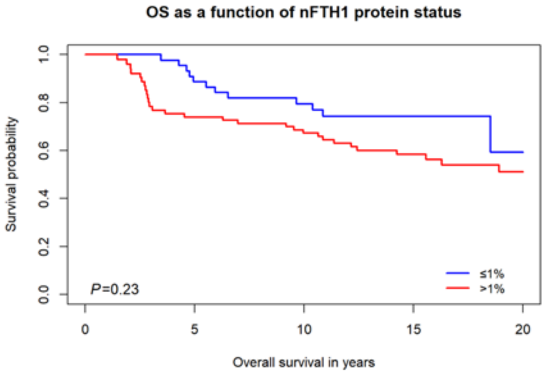

C

	0	2	4	6	8	10	12	14	16	18	20
nFTH1≤1%	31	41	45	38	39	31	19	17	9	6	2
nFTH1>1%	15	49	56	57	55	49	44	36	27	19	16

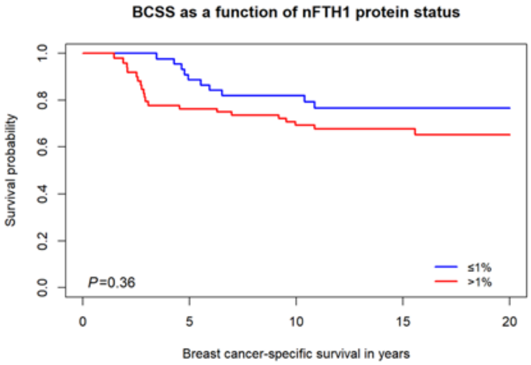

D

	0	2	4	6	8	10	12	14	16	18	20
nFTH1≤1%	31	41	45	38	39	31	18	16	8	5	2
nFTH1>1%	15	47	55	56	54	48	42	34	26	19	16

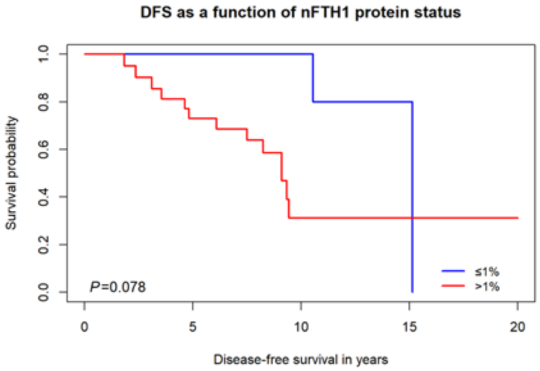

E

	0	2	4	6	8	10	12	14	16	18	20
nFTH1≤1%	3	8	7	7	6	5	4	2	0	0	0
nFTH1>1%	20	20	20	16	14	5	5	2	1	1	1

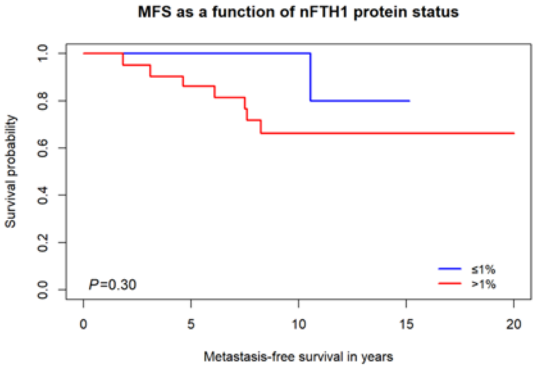

F

	0	2	4	6	8	10	12	14	16	18	20
nFTH1≤1%	3	8	7	7	6	5	4	2	0	0	0
nFTH1>1%	20	20	22	18	15	7	7	3	2	2	2

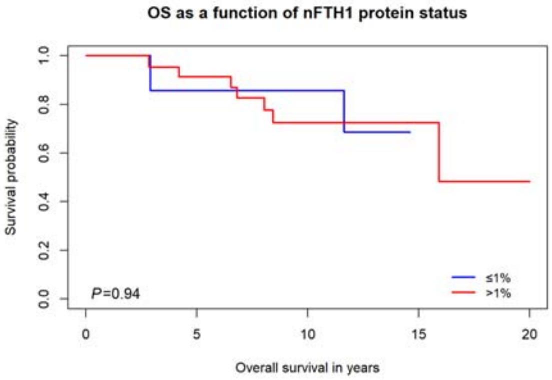

G

	0	2	4	6	8	10	12	14	16	18	20
nFTH1≤1%	2	7	6	6	5	5	4	1	0	0	0
nFTH1>1%	21	21	24	22	18	10	10	4	2	2	2

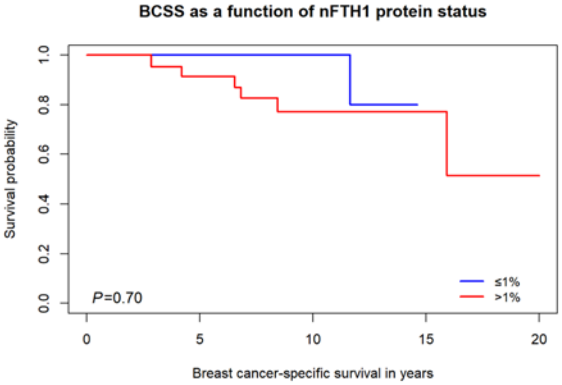

H

	0	2	4	6	8	10	12	14	16	18	20
nFTH1≤1%	2	6	6	6	5	5	4	1	0	0	0
nFTH1>1%	21	21	24	22	18	10	10	4	2	2	2

Supplementary Figure S1. Kaplan-Meier survival curves of *BRCA1* and *BRCA2* mutation carriers stratified by nFTH1 expression. A, disease-free survival in *BRCA1* mutation carriers; B, metastasis-free survival in *BRCA1* mutation carriers; C, overall survival in *BRCA1* mutation carriers; D, breast cancer-specific survival in *BRCA1* mutation carriers; E, disease-free survival in *BRCA2* mutation carriers; F, metastasis-free survival in *BRCA2* mutation carriers; G, overall survival in *BRCA2* mutation carriers; H, breast cancer-specific survival in *BRCA2* mutation carriers. *P*-values are from the logrank test. The number of persons at risk per timepoint are indicated below each graph.
